# Certain vs. uncertain actionable secondary findings in a cohort of 500 Lebanese participants: What to report to the patient?

**DOI:** 10.1371/journal.pone.0327471

**Published:** 2025-07-18

**Authors:** Eileen Marie Hanna, Cybel Mehawej, Yazid Hoblos, Kelven Rahy, Andre Megarbane, Eliane Chouery

**Affiliations:** 1 Department of Computer Science and Mathematics, Lebanese American University, Byblos, Lebanon; 2 Department of Human Genetics, Gilbert and Rose-Marie Chagoury School of Medicine, Lebanese American University, Byblos, Lebanon; 3 Gilbert and Rose-Marie Chagoury School of Medicine, Lebanese American University, Byblos, Lebanon; 4 Institut Jérôme Lejeune, Paris, France; University of Miami Miller School of Medicine: University of Miami School of Medicine, UNITED STATES OF AMERICA

## Abstract

Advances in next-generation sequencing enabled its integration into genetic diagnosis and have led to the uncovering of secondary findings. In this paper, we analyzed 500 Lebanese participants for pathogenic and likely-pathogenic variants in 81 recommended genes listed by the American College of Medical Genetics (ACMG). In this retrospective study, 500 individuals seeking genetic diagnosis through Exome Sequencing were included. Variants were analyzed and their pathogenicity assessed based on ACMG/AMP criteria and ClinVar. Secondary findings were identified in 16.8% of cases based on ACMG/AMP criteria, which decreased to 6% when relying on ClinVar. Dominant cardiovascular disease variants were predominant, constituting 6.6% based on ACMG/AMP assessments and 2% according to ClinVar. Additionally, using ACMG/AMP criteria, dominant oncogenic variants were identified in 4.2% of individuals, while recessive pathogenic variants were found in 4.8%. In contrast, ClinVar-based analysis reported these variants in 1% and 2.6% of the cohort, respectively. The high discordance between ACMG/AMP and ClinVar classifications (16.8% vs. 6%) underscores ethical dilemmas in deciding which criteria to prioritize for patient disclosure. Indeed, the absence of ACMG-classified pathogenic or likely pathogenic variants in ClinVar complicates reporting due to a lack of evidence linking them to disease in other individuals. Finally, the significant discrepancy between ACMG/AMP and ClinVar classifications emphasizes the urgent need to harmonize variant databases and update ClinVar entries, particularly for understudied populations such as the Lebanese cohort.

## Introduction

Advances in next-generation sequencing (NGS) techniques and bioinformatics have made genomic sequencing a clinical diagnostic tool for genetic and rare diseases. Although genome sequencing (GS) was initially employed, the focus on coding regions led to the development of exome sequencing (ES) as a more cost-effective alternative [1]. The widespread use of ES and GS has raised the issue of secondary findings; genetic variants unrelated to the primary indication for testing but with potential clinical significance. To address this, the American College of Medical Genetics (ACMG) released recommendations for analyzing and reporting such findings.

Since its initial publication in 2013, the ACMG list of actionable genes has undergone several revisions, expanding from 56 to 81 genes in its latest version (v3.2) [2–7]. These updates reflect the growing understanding of clinically significant variants and their implications for patient care.

While numerous studies have reported the prevalence of pathogenic (P) and likely-pathogenic (LP) secondary findings across diverse populations [8–12], data from underrepresented groups, such as Middle Eastern cohorts, remain scarce. Understanding these findings is critical for identifying at-risk individuals and implementing preventive measures.

In a previous work, we reported the frequency of P and LP variants in 56 actionable genes in the Lebanese population [13]. In this paper, a new cohort consisting of 500 unrelated Lebanese participants presenting with a wide spectrum of genetic disorders who were referred to the Department of Human Genetics at the Lebanese American University Gilbert and Rose-Marie Chagoury School of Medicine was assessed for secondary findings based on the latest revision of the ACMG (SF v3.2) list consisting of 81 genes.

## Results

### General findings

Over the last 3 years, 500 patients, seeking genetic diagnosis by ES, were referred to our institution. The studied cohort primarily comprised pediatric patients, with a minority being young adults seeking genetic counseling prior to marriage. All those patients, or their legal guardians, wished to receive information on actionable secondary findings, unrelated to the condition for which genetic testing was performed.

Following the most recent version of the ACMG guidelines, P and LP variants in 81 actionable genes were analyzed. This led to the identification of 9632 variants. After applying quality-filtering criteria of a minimum depth (DP) of 15x and a minimum allele balance (AB) of 30%, the number of detected variants was reduced to 8596. S1 Fig highlights the frequency distribution of both DP and AB of all 9632 identified variants.

The pathogenicity of the filtered variants was then examined based on the guidelines for the interpretation of sequence variants established by the ACMG and the Association for Molecular Pathology (ACMG/AMP) [4] as well as on ClinVar database [14], and taking into consideration ClinGen Variant Curation Expert Panels (VCEPs). ACMG/AMP classification, was initially attributed to each variant, using several tools (such as InterVar, VSClinical, VarSome, Franklin); then confirmed by a manual verification to ensure accuracy and reliability. This enabled the identification of 63 P/LP variants in 84 individuals (Table 1 and S1 Table), while ClinVar identified 20 P/LP variants in 30 participants (Table 1). As expected, a statistically significant association between the two classification systems was observed (data not shown). Indeed, all P and LP variants present in ClinVar were also classified as P or LP by the ACMG/AMP guidelines (Table 1).

**Table 1:** Complete list of all the pathogenic (P) and likely pathogenic (LP) variants of the actionable ACMG SF v3.2 genes identified in our cohort based on the ACMG/AMP guidelines. The variants that are found to be pathogenic or likely pathogenic in ClinVar are highlighted in the rightmost column.

Genes	Gene MIM	Disease MIM	Inheritance Mode	Identifier	cDNA position	Protein change	DNA change	Num alleles	ACMG/ AMP Guidelines	ACMG/AMP Categories	ClinVar (number of stars)/number of submitters
***APC***	611731	175100	AD	NM_000038.5	c.2856delC	p.Lys953fs	112174145 GC- > G	1	LP	PVS1_Strong; PM2_Moderate	–
***ATP7B***	606882	277900	AR	NM_000053.3	c.1285 + 5G > T		52548066 C- > A	3	LP	PM3_Very strong; PM2_Moderate; PP3_Moderate	–
	606882	277900	AR	NM_000053.3	c.3865G > A	p.Asp1289Asn	52511650 C- > T	1	LP	PM2_Moderate; PM1_Moderate; PP2_Supporting; PP3_Supporting	–
	606882	277900	AR	NM_000053.3	c.19_20delCA	p.Gln7fs	52585453 CTG- > C	1	P	PVS1_Very Strong; PM2_Moderate; PM3_Strong	Conflicting (*)/12
	606882	277900	AR	NM_000053.3	c.3207C > A	p.His1069Gln	52518281 G- > T	2	P	PS3_Supporting; PM1_Moderate; PM2_Moderate; PM3_very Strong; PP1_Supporting; PP2_Supporting; PP3_Supporting	P (**)/39
	606882	277900	AR	NM_000053.3	c.3199A > G	p.Ser1067Gly	52518289 T- > C	1	LP	PM1_Moderate; PM2_Moderate; PP2_Supporting; PP3_Moderate	Uncertain significance (*)/1
***BRCA2***	600185	612555	AD	NM_000059.3	c.1237delC	p.Leu413fs	32906848 AC- > A	1	LP	PVS1_Very Strong; PM2_Moderate	P (***)/4
***CACNA1S***	114208	601887	AD	NM_000069.2	c.3256C > T	p.Arg1086Cys	201029944 G- > A	1	LP	PM2_Moderate; PM5_Moderate; PP3_Strong	Uncertain significance (**)/11
***DES***	125660	604765 601419	AD	NM_001927.3	c.854C > T	p.Ala285Val	220285335 C- > T	1	LP	PS4_Moderate; PS3_Supporting; PM2_Moderate; PP2_Supporting; PP3_Supporting	Conflicting (*)/2
***DSG2***	125671	610193	AD	NM_001943.4	c.3036_3037insG	p.Tyr1013fs	29126385 T- > TG	2	P	PVS1_Strong; PS4_strong;PM2_Moderate	LP (**)/2
***FBN1***	134797	154700	AD	NM_000138.4	c.4588C > T	p.Arg1530Cys	48760294 G- > A	1	LP	PS4_Strong; PM1_Moderate; PM2_Moderate; PP1_Supporting; PP2_Supporting; PP3_Supporting	LP; P (**)/15
***FLNC***	102565	617047 609524	AD	NM_001458.4	c.7561G > A	p.Gly2521Ser	128496975 G- > A	1	LP	PM2_Moderate; PP3_Very strong	Uncertain significance (**)/3
***GAA***	606800	232300	AR	NM_000152.4	c.1828G > A	p.Ala610Thr	78086450 G- > A	2	LP	PM1_Moderate; PM2_Moderate; PP3_Moderate	Uncertain significance (***)/9
	606800	232300	AR	NM_000152.4	c.1064T > C	p.Leu355Pro	78082197 T- > C	1	P	PS3_Supporting; PM3_Very Strong; PM2_Moderate; PM1_Moderate; PP3_Supporting	P (***)/10
	606800	232300	AR	NM_000152.4	c.1958C > A	p.Thr653Asn	78086744 C- > A	1	LP	PM3_Strong; PM2_Moderate; PM1_Moderate; PP3_Supporting	Conflicting (*)/3
	606800	232300	AR	NM_000152.4	c.1396dupG	p.Val466fs	78083807 A- > AG	1	LP	PVS1_Very Strong; PM2_Moderate	P(**)
***HFE***	613609	235200	AR	NM_000410.3	c.845G > A	p.Cys282Tyr	26093141 G- > A	2	LP	PS3_Strong; PP3_Moderate	P(**)/47
	613609	235200	AR	NM_000410.3	c.187C > G	p.His63Asp	26091179 C- > G	135	P	PS3_Strong; PS1_Strong; PM1_Supporting	P; LP (**)/50
***KCNH2***	152427	613688	AD	NM_000238.3	c.1469C > T	p.Ala490Val	150649601 G- > A	1	LP	PM2_Moderate; PM5_Moderate; PP2_Supporting; PP3_Strong	Conflicting (*)/3
	152427	613688	AD	NM_000238.3	c.2086C > T	p.Arg696Cys	150648068 G- > A	1	LP	PM2_Moderate; PP2_Supporting; PP3_Strong	Uncertain significance (*)/2
	152427	613688	AD	NM_000238.3	c.2764C > T	p.Arg922Trp	150644895 G- > A	1	LP	PM2_Moderate; PP3_Strong; PP2_Supporting	Uncertain significance (**)/4
***KCNQ1***	607542	192500	AD	NM_000218.2	c.877C > T	p.Arg293Cys	2594172 C- > T	1	LP	PM2_Moderate; PM1_Moderate; PP3_Supporting; PP2_Supporting	Conflicting (*)/11
	607542	192500	AD	NM_000218.2	c.514G > A	p.Val172Met	2591894 G- > A	1	LP	PM1_Moderate; PM2_Moderate; PP3_Supporting; PP2_Supporting	Conflicting (*)/11
***LDLR***	606945	143890	AD	NM_000527.4	c.2043C > A	p.Cys681*	11231101 C- > A	6	P	PVS1_Very Strong; PS4_Moderate; PM2_Moderate	P; LP(**)/31
	606945	143890	AD	NM_000527.4	c.859G > A	p.Gly287Ser	11218109 G- > A	2	LP	PM2_Moderate; PM1_Moderate; PP3_Moderate	Uncertain significance (***)/10
***LMNA***	150330	115200	AD	NM_170707.3	c.1196G > A	p.Arg399His	156106043 G- > A	1	LP	PM2_Moderate; PM1_Moderate; PP2_Moderate	Uncertain significance (**)/7
	150330	115200	AD	NM_170707.3	c.1357C > T	p.Arg453Trp	156106204 C- > T	1	P	PS2_Strong; PS3_Supporting; PM1_Moderate; PM2_Moderate; PP3_Supporting; PP2_Supporting	P; LP (**)/18
	150330	115200	AD	NM_170707.3	c.808A > C	p.Lys270Gln	156104764 A- > C	3	LP	PM1_Moderate; PM2_Moderate; PP3_Supporting; PP2_Supporting	Uncertain significance (**)/3
***MLH1***	120436	609310	AD	NM_000249.3	c.91_92delGCinsTG	p.Ala31Cys	37035129 GC- > TG	4	LP	PS3_Supporting; PM5_Strong; PM2_supporting	Conflicting (*)/13
***MSH2***	609309	120435	AD	NM_000251.2	c.2012A > G	p.Asn671Ser	47703512 A- > G	1	P	PM1_Strong; PM2_Supporting; PP3_Strong	Conflicting (*)/4
	609309	120435	AD	NM_000251.2	c.1045C > G	p.Pro349Ala	47643537 C- > G	2	LP	PM5_Moderate; PM2_Moderate; PP3_Strong	Conflicting (*)
***MUTYH***	604933	608456	AR	NM_001128425.1	c.1187G > A	p.Gly396Asp	45797228 C- > T	3	LP	PM2_Moderate; PM5_Moderate; PP3_Strong	P; LP (**)/70
	604933	608456	AR	NM_001128425.1	c.536A > G	p.Tyr179Cys	45798475 T- > C	1	P	PS3_Supporting; PM1_Moderate; PM2_Moderate; PM3_Very Strong; PP1_Supporting; PP3_Moderate	LP; P (**)/62
	604933	608456	AR	NM_001128425.1	c.933_933 + 1delAG	p.Val312fs	45797836 ACT- > A	1	P	PVS1_Very strong; PM2_Moderate; PP5_Supporting	P; LP (**)/3
***MYBPC3***	600958	115197	AD	NM_000256.3	c.1591G > C	p.Gly531Arg	47364162 C- > G	1	P	PS1_Strong; PS3_Supporting; PS4_Moderate; PM2_Moderate; PM5_Supporting; PP1_Supporting; PP3_Moderate	Conflicting (*)
***MYH7***	160760	192600 613426	AD	NM_000257.3	c.2614G > A	p.Glu872Lys	23894043 C- > T	1	LP	PM1_Strong; PM2_Moderate; PP3_Supporting	–
***OTC***	300461	311250	XL	NM_000531.5	c.622G > A	p.Ala208Thr	38262952 G- > A	1	P	PM1_Moderate; PM2_Moderate; PM3_Very Strong; PP1_Supporting; PP2_Supporting; PP3_Supporting	P (**)/9
	300461	311250	XL	NM_000531.5	c.929_931delAAG	p.Glu310del	38271170 CAGA- > C	1	P	PS2_Strong; PM1_Moderate; PM2_Moderate; PM4_Moderate	P (**)/2
***PALB2***	610355	114480	AD	NM_024675.3	c.3350 + 4A > G		23619181 T- > C	1	LP	PS4_Strong; PM2_Supporting; PM3_Very Strong	LP (***)/13
***PKP2***	602861	609040	AD	NM_004572.3	c.1470delG	p.Ile491fs	32996155 TC- > T	1	LP	PVS1_Very strong; PM2_Moderate	–
***PMS2***	600259	614337	AD	NM_001322015.1	c.11C > G	p.Ser4*	6042301 G- > C	2	LP	PVS1_Strong; PM2_Moderate	Conflicting (*)
***RET***	164761	155240 171400 162300	AD	NM_020975.4	c.2370G > T	p.Leu790Phe	43613906 G- > T	1	P	PS1_Strong; PS3_Supporting; PS4_Moderate; PM1_Moderate; PM2_Moderate; PP1_Supporting; PP3_Supporting	P; LP(**)/9
***RPE65***	180069	204100 613794	AR	NM_000329.2	c.118G > A	p.Gly40Ser	68912520 C- > T	1	P	PS3_Supporting; PS4_Strong; PM1_Moderate; PM2_Moderate; PP1_Supporting; PP3_Moderate	P (***)/10
	180069	204100 613794	AR	NM_000329.2	c.1451G > A	p.Gly484Asp	68895610 C- > T	1	LP	PM2_Moderate; PM5_Moderate; PP3_Strong	LP (***)/10
***RYR1***	180901	145600	AD	NM_000540.2	c.1099C > T	p.Arg367Trp	38939430 C- > T	2	LP	PS3_Strong; PM5_Supporting; PM1_Supporting	Conflicting (*)/5
	180901	145600	AD	NM_000540.2	c.1859T > G	p.Ile620Ser	38948204 T- > G	1	LP	PM5_Moderate; PP3_Strong	Uncertain significance (*)/1
***SCN5A***	600163	603830 601144 601154	AD	NM_001099404.1	c.2423G > A	p.Arg808His	38628904 C- > T	1	LP	PM2_Moderate; PM5_Moderate; PP3_Strong	Uncertain significance (**)/3
	600163	603830 601144 601154	AD	NM_001099404.1	c.1700T > A	p.Leu567Gln	38645393 A- > T	1	LP	PM1_Moderate; PM2_Strong	Uncertain significance (**)/10
***SDHB***	185470	115310 171300	AD	NM_003000.2	c.529C > A	p.Arg177Ser	17354255 G- > T	1	LP	PM1_Moderate; PM2_Moderate; PP3_Moderate	Uncertain significance (**)/2
	185470	115310 171300	AD	NM_003000.2	c.289A > T	p.Ile97Phe	17355229 T- > A	1	LP	PM1_Moderate; PM2_Moderate; PP2_Supporting_PP3_Supporting	Conflicting (*)/4
***SMAD3***	603109	613795	AD	NM_001145103.1	c.72delG	p.Arg25fs	67430432 TG- > T	1	LP	PVS1_Strong; PM2_Moderate	Uncertain significance (*)/1
***SMAD4***	600993	174900 175050	AD	NM_005359.5	c.1498A > G	p.Ile500Val	48604676 A- > G	1	P	PS2_Strong; PS3_Supporting; PM1_Supporting; PM2_Moderate; PP2_Supporting; PP3_Supporting	P (**)/38
	600993	174900 175050	AD	NM_005359.5	c.3G > A	p.Met1?	48573419 G- > A	1	LP	PVS1_Moderate; PM2_Moderate	Uncertain significance (*)/1
***TGFBR2***	190182	610168	AD	NM_001024847.2	c.1138G > C	p.Ala380Pro	30713738 G- > C	1	P	PS4_Strong; PM1_Moderate; PM2_Moderate; PP2_Strong; PP3_Strong	Conflicting (*)/4
***TP53***	191170	151623	AD	NM_000546.5	c.328C > T	p.Arg110Cys	7579359 G- > A	1	LP	PM1_Strong; PM2_Moderate	Uncertain significance (**)/12
	191170	151623	AD	NM_000546.5	c.461G > A	p.Gly154Asp	7578469 C- > T	1	LP	PM1_Moderate; PM2_Moderate; PP3_Moderate; BS3_Supporting	Uncertain significance (***)
***TRDN***	603283	615441	AR	NM_006073.3	c.1805-1G > T		123581784 C- > A	1	LP	PVS1_Moderate; PM2_Moderate	–
	603283	615441	AR	NM_006073.3	c.1537 + 1G > A		123600200 C- > T	1	LP	PVS1_Strong; PM2_Moderate	Uncertain significance (**)
***TSC2***	191092	613254	AD	NM_000548.4	c.2546-1G > A		2125799 G- > A	1	P	PVS1_Very strong; PS2_Strong; PM2_Moderate	P (*)/2
	191092	613254	AD	NM_000548.4	c.2T > C	p.Met1?	2098618 T- > C	1	P	PVS1_Moderate; PS1_Strong; PM2_Moderate	Uncertain significance (**)
***TTN***	188840	604145	AD	NM_001267550.2	c.22528 + 1_22528+ 17delGTTTGTTCACACGTCAC		179586968 AGTGACGTGT GAACAAAC- > A	1	LP	PVS1_Strong; PM2_Moderate	–
***VHL***	608537	193300	AD	NM_000551.3	c.373C > T	p.His125Tyr	10188230 C- > T	1	LP	PM1_Moderate; PM2_Strong	Conflicting (*)/8
	608537	193300	AD	NM_000551.3	c.242C > A	p.Pro81Gln	10183773 C- > A	1	LP	PM1_Strong; PM2_Moderate	Conflicting (*)/3

A total of 63 P and LP variants classified through ACMG/AMP guidelines were found in 36 different genes out of the 81. From these, 43 variants located in 29 genes involved in autosomal dominant (AD) diseases were found in 58 participants (11.6% of our cohort), 18 variants in 6 genes involved in autosomal recessive (AR) diseases in 24 individuals (4.8% of our cohort), and two in a gene involved in a X-linked (XL) disease in two participants (0.4% of our cohort) (Table 1).

The 20 P/LP variants identified through ClinVar are located in 15 genes: 9 genes involved in diseases with AD inheritance, 5 genes linked to AR diseases, and one XL disease (S2 Fig). Missense and frame-shift variants were the most common types of identified variants, accounting for 46 and 8, respectively (S3 Fig).

### Dominant medically actionable diseases

**Fig 1:**
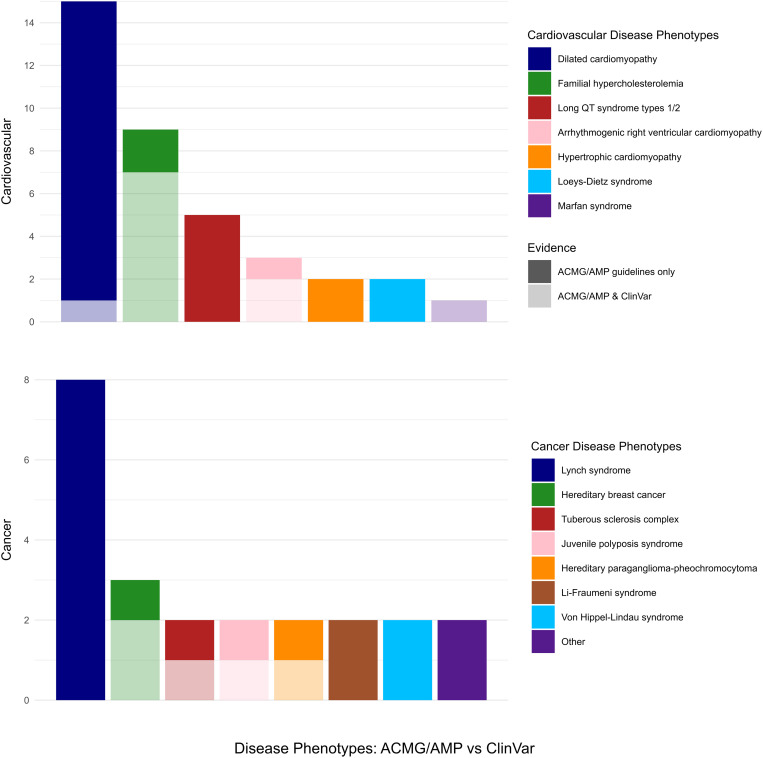
Summary of actionable gene frequencies in autosomal dominant (AD) disorders. Each bar represents the number of participants per disease. The upper portion of each bar indicates the frequency of actionable genes based on ACMG criteria, while the lower portion reflects frequencies according to ClinVar data.

Among the dominant medically actionable variants, those associated with cardiovascular diseases were found in 33 participants out of the 84 identified individuals with secondary findings (39%) and accounting for 6.6% of our cohort. Furthermore, variants in the *LDLR* gene (NM_000527.4), linked to familial hypercholesterolemia (FH) were found in 8 individuals. ClinVar helped to discard one of the variants in the *LDLR* gene as having conflicting interpretations of pathogenicity. Two variants in genes associated with long QT syndrome types 1/2 were found in five participants. Similarly, two variants in genes implicated in hypertrophic cardiomyopathy and two others in genes implicated in Loeys-Dietz syndrome were identified (Fig 1).

Carcinogenic dominant variants were detected in 21 individuals, representing 25% of the participants with secondary findings and 4.2% of our cohort. Of these, one individual was found to carry a variant in *BRCA2* (NM_000059.3), and another one carried a variant in *PALB2* (NM_024675.3); both genes being associated with increased risk for breast cancer. On another hand, three genes (*TSC2, SMAD4* and *SDHB*) were found to harbor 2 variants each. One of the variants associated with each of these conditions was found in ClinVar to be of uncertain significance. In addition, four P/LP variants were found in genes associated with Lynch syndrome in eight participants. However, none of these was listed as P or LP in ClinVar. Similarly, variants implicated in Li-Fraumeni syndrome, Von Hippel-Lindau syndrome, and familial adenomatous polyposis were identified in five individuals, but none of them was found to be P or LP in ClinVar (Fig 1).

### Recessive medically actionable diseases

**Fig 2:**
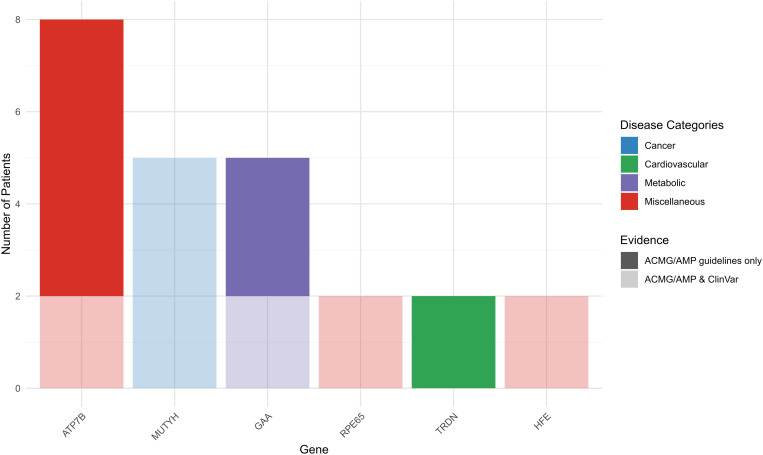
Summary of the actionable genes’ frequencies in diseases with AR inheritance. The upper portion of each bar indicates the frequency of actionable genes based on ACMG criteria, while the lower portion reflects frequencies according to ClinVar data. Gene MUTYH corresponds to the MUTYH-associated polyposis phenotype, in the Cancer category. Gene *TRDN* is related to Long QT syndrome, in the Cardiovascular category. Gene *GAA* corresponds to Pompe disease, in the metabolic category. Genes *ATP7B*, *RPE65*, and *HFE* correspond to Wilson disease, *RPE65*-related retinopathy, and Hereditary Hemochromatosis in the miscellaneous category, respectively.

Recessive pathogenic variants in six actionable genes were detected at a heterozygous state in 24 individuals, representing 28.6% of the participants with reportable secondary findings and 4.8% of our cohort. Five different variants in the *ATP7B* gene (NM_000053.3) implicated in Wilson disease were identified in eight participants. Only one of these variants was listed as P in ClinVar. Similarly, four variants in the *GAA* gene (NM_000152.4) implicated in Pompe disease were identified in five individuals, but two of them were found as P in ClinVar. In addition, three variants in the *MUTYH* gene (NM_001048174.1) carried by five participants were found to be P or LP by both ACMG/AMP guidelines and ClinVar (Fig 2).

## Discussion

Five hundred individuals referred to our institution and seeking genetic diagnosis through ES were included in this study. Generated data was screened for secondary findings following the most recent ACMG guidelines and taking into account VCEPs recommendations. Identifying and reporting these findings is of high importance. Indeed, this enables to reduce associated morbidity and mortality in some cases and helps in genetic counseling and reproductive planning approaches [15,16].

### Ethical and practical challenges in reporting secondary findings

Reporting of secondary findings encounters various ethical and practical challenges such as the right of patients to choose to decline knowing such incidental findings, the additional work for data analysis and reporting as well as the additional costs [17]. On another hand, challenges exist in the interpretation of these secondary findings, encompassing the risk of false positives in reporting results to patients and potential complexities arising from incomplete penetrance [3]. For instance, if a secondary finding reveals a genetic variant associated with increased susceptibility to develop a certain cancer, the individual may experience anxiety and undergo unnecessary or medical interventions, even though he/she will never develop the disease. Similarly, their family members may also be unnecessarily concerned about their own risk.

Moreover, the definitions of actionability might vary between ACMG/AMP and non-ACMG recommendations, such as those from ClinGen and ESHG [18,19]. Considering this lack of standardization, a review of a large cohort of stakeholders’ views on secondary findings emphasized the importance of bidirectional communication and dynamic consent forms [20]. This allows the patients to make informed decisions while being able to change their preferences. Accordingly, most testing laboratories’ policies and professional recommendations are moving in the direction of emphasizing the clarity of the policies and consent forms for both the analysis and the report of secondary findings. Moreover, it is important to note that the use of some ACMG criteria remains debatable. For instance, the use of the PM2 criterion, while helpful, has limitations, particularly when evaluating actionable genes. This underscores the necessity of integrating additional lines of evidence to ensure accurate variant classification, emphasizing a more comprehensive approach rather than relying solely on population frequency data.

### Comparison of our findings with previously published data

Our analysis based on ACMG/AMP guidelines for variants classifications, showed a frequency of secondary findings of 16.8% in our cohort. Alternatively, relying exclusively on the clinically confirmed verdicts of ClinVar narrows the number of P and LP variants to 20 variants in 30 participants, accounting for 6% of our cohort (and representing 31.7% of all variants predicted to be P/LP by the ACMG/AMP guidelines). The large variability in the percentages of secondary findings based on ACMG/AMP and ClinVar are not surprising considering that ClinVar is more stringent since it does not take into account pathogenicity scores but relies mostly on data reported in patients [21]. The frequency identified in our cohort based on ClinVar (6%) aligns with previously reported rates of secondary findings, which range from 1% to 9% in similar studies [22]. Indeed, a frequency of 7% in a Korean cohort of 100 control subjects and 6% in 96 Korean patients affected with different diseases was identified [8]. Other reports found a rate of 3.5% in a large cohort of Qataris [23] and 3.6% for a cohort of 836 non-obstructive azoospermia cases from a large international study [10]; a similar frequency of 3.2% was reported for a cohort of 789 unaffected parents of children with developmental delay and intellectual disability [11]; lower frequencies were reported in a GS study for a cohort of 954 East Asians reporting a frequency of 2.5% [9] as well as an ES study for 1640 anonymized healthy Dutch individuals reporting a frequency of 2.7% [22]; and a frequency as low as 1.5% was reported for a large cohort of 2480 Japanese cancer patients [24]. The discrepancies in the reported percentages are not surprising, considering the different guidelines and lists of genes adopted such as: different versions of the ACMG minimal list, the varying sizes of cohorts and the differences in study designs and the medical status of the included individuals, the variations at the level of the sequencing technologies, the incomplete coverage, and the quality of the reads. Moreover, the difficulty in the interpretation of variants is a main factor leading to such discrepancies. The confidence in the pathogenicity established through clinical evidence varies significantly between variants, with many variants remaining of uncertain significance, and the lists of variants to be examined being constantly updated. The interpretation of copy number variants (CNVs) adds another challenge. In our analysis, only Single Nucleotide Variants (SNVs) detected by ES were considered.

### Analysis of dominant cardiovascular diseases

Variants associated with dominant cardiovascular diseases were the most prevalent in our cohort, identified with a percentage of 6.6% based on ACMG/AMP assessments and in 2% according to those of ClinVar. This is in accordance with our previous report [13] in which AD cardiovascular variants were also found to be the most prevalent with an occurrence rate of 2.2%. Moreover, in our previous cohort, P/LP variants were identified at a frequency of 1:70 patients in genes associated with LQTS. Similarly, in this study, 3 variants in the *KCNH2* gene and 2 variants in the *KCNQ1* gene were found in 5 patients. Additionally, 2 variants in the *SCN5A* gene associated with both dilated cardiomyopathy and LQTS type 3 were found in 2 other patients leading to a total of 7:500 (~1:70). In accordance with Jalkh et al., a general pattern towards increased frequency of LQTS in Lebanon is noted compared to its global frequency of 1:2500; which urges the necessity of further studying and investigating these genes in the Lebanese population at large.

Variants linked to FH were found in 1.6% of our cohort. This number is reduced to 1.2% (1:80) based on the ClinVar verdicts which is similar to our previous report, casting doubt on the pathogenicity of one of the 2 variants in the *LDLR* gene. As discussed before, the frequency of the p.Cys681* in *LDLR* in the Lebanese population has been previously highlighted and presumed to be due to a founder effect [25]; this P variant being frequent and accounts for 81.5% of FH cases in Lebanon. The prevalence of FH in the worldwide population is reported to be 0.32% (1:313) [26], thus highlighting the more than 4-fold increase in its prevalence in the Lebanese population. Therefore, preventive measures must be taken to face this high prevalence of FH, and its molecular diagnosis should be facilitated to prevent deaths from coronary heart events.

### Analysis of oncogenic dominant diseases

Oncogenic dominant variants were observed in 4.2% of our cohort, of whom 2 individuals presented with P variants in *BRCA2* and *PALB2*, both being linked to breast cancer and accounting together for 0.4% of our cohort.

Breast cancer was reported to be the most prevalent type of cancer in the Lebanese population, accounting for 37% of cancer cases among females and 20% of all cancer cases, with the incidence rate averaging 91.7 per 100,000 between 2005 and 2015 [27]. The prevalence of *BRCA1/2* mutations is highly variable between different populations and ethnicities. A review of multiple reports suggests that the frequency of P variants in *BRCA1/2* is estimated to be 1:400 in some populations, while it is as high as 1:200 in others [28]. Generally, *BRCA2* pathogenic variants are reported to have a higher incidence rate than those of *BRCA1*, and the rate of those of *PALB2* is reported to be lower [11,29,30]. Considering the high variability in the rates of *BRCA1/2* across populations along with their values in our previous and current cohort (1:140 and 1:500 respectively), including more individuals in the tested cohort must be considered to better assess the prevalence of these mutations in the Lebanese population.

In addition, variants in 3 genes associated with Lynch syndrome were found in 8 patients. However, ClinVar revealed one of them to be LP and the others to have conflicting interpretations of pathogenicity. Of the 17 variants associated with AD carcinogenic conditions identified by ACMG/AMP, only 5 variants (1% from our cohort) were confirmed as P or LP in ClinVar (Fig 1); thus, stressing the complexity of variant interpretation in genetic counseling.

### Analysis of recessive diseases

Recessive pathogenic variants were found in 4.8% of our cohort. This number is further reduced to 2.6% based on ClinVar interpretations of pathogenicity. The frequencies of P variants in *MUTYH* and *ATP7B* remained almost the same as in our previous cohort. Indeed, the frequency of *MUTYH* variants went from 1:90–1:100 and that of *ATP7B* variants increased from 1:70–1:60. Excluding the *ATP7B* variants that were not confirmed to be P in ClinVar reduces significantly the frequency of *ATP7B* variants to 1:250. Frequencies of P/LP variants in both *MUTYH* and *ATP7B* in our previous cohort were shown to be in accordance with the general population rates and they remain so. It is worth noting that a specific variant in the *MUTYH* gene (NM_001128425:c.1187G > A; p.Gly396Asp) was found in 2 participants in the previous cohort and found in 3 participants in the current one, suggesting that it may be exceptionally prevalent in the Lebanese population.

On another hand, the frequency of P/LP variants in *GAA* is reported to be 1.3% (1:77) in the general population [31] correlating with the frequency found in our cohort (1:100). Two P variants in the *RPE65* gene, carried each by one individual, were confirmed as P in both ACMG/AMP guidelines and ClinVar. While the frequency of the *RPE65* pathogenic variants was reported to be 0.10% for East Asians and 0.06% for Koreans [32] a frequency of 1.87% was reported in a Chinese cohort of 1069 genetically diagnosed patients [33]. Thus, the 0.4% frequency found in our cohort seems to fall within the normal ranges. Finally, 2 P variants in the *HFE* gene were identified in our cohort but following the ACMG recommendations only homozygous occurrences of the c.845G > A; p.Cys282Tyr variant were considered. It must be noted that the p.Cys282Tyr variant, which is specifically highlighted to be reported in the ACMG minimal list of genes, was also identified as pathogenic in ClinVar.

According to ACMG guidelines, variants involved in recessive diseases are not reported unless they are present at homozygous or compound heterozygous states in the patient. However, considering the high rate of consanguineous marriages in the Lebanese population (35.5% of marriages), reporting these heterozygous occurrences is of high importance [34]. Indeed, the practice of consanguineous marriages elevates the risk of inheriting autosomal recessive disorders due to the increased likelihood of sharing identical genetic material between closely related individuals. Consequently, reporting these heterozygous occurrences is essential for genetic counseling, familial risk assessment, and public health interventions.

### Summary and conclusion

In summary, following the ACMG guidelines, informed consent was obtained from 500 Lebanese participants to analyze and report the secondary findings of their ES results. This secondary analysis is highly beneficial in many ways. First, it allows preventive and/or therapeutic measures to be taken on the part of the patients with medically actionable P variants and their relatives. Second, it supports reproductive planning and implementing neonatal screening tests, especially considering the high rate of consanguineous marriages. Third, the statistical analysis of the ES secondary findings at the level of our cohort helps achieve a better understanding of the P variants that appear to be prevalent in our population. As a result, we are able to propose preventive measures, such as facilitated testing for some conditions in cases of previous familial history, and potentially identify founder effects or speculate upon the origin of certain variants and their evolution in our community.

Most importantly, this study shows the complexity of data interpretation depending on the adopted strategy. Indeed, reporting indisputable variants, such as those reported in ClinVar by several submitters, and already associated with clinical manifestations in several patients, is considered more confident than delivering questionable information related to newly classified genetic variants. That being said, clinical geneticists should be aware that the credibility of the class attributed to each variant on ClinVar can be influenced by various factors, such as the number of submissions supporting a particular classification and the quality of the evidence provided by those submissions. Variants with multiple submissions concurring on their classification are generally considered to have higher confidence levels. However, conflicting interpretations in ClinVar, following different divergent submissions, can also complicate the analysis and necessitate thus deep analysis in order to determine whether the variant should be disregarded due to lack of conclusive data. Moreover, rare variants specifically those identified in isolated populations are less likely to be present in ClinVar due to their rarity. It is thus crucial for clinicians and researchers working with these populations to submit their findings to ClinVar. This ensures that these rare variants are available for the wider research community, facilitating a better understanding of their clinical implications.

In our clinical practice, we typically rely on ClinVar as a reference during genetic counseling. However, all variants classified as P or LP according to ACMG guidelines are reported to patients with great care. This process includes comprehensive genetic counseling that takes into account the patient’s family history, and when appropriate, clinical re-evaluation. In certain cases, we also recommend regular follow-up with patients after one to two years to allow time for potential reclassification of variants, ensuring that patients receive the most accurate and up-to-date information regarding their genetic findings.

In conclusion, gathering the efforts of scientists to update genetic public databases becomes crucial. This is essential for enhancing the utility of public databases, their accuracy, and relevance in clinical practice and research. For instance, guidance from the VCEPs for many genes underscores this collective effort. On another hand, diversity in genetic data collection is also essential to contribute to more equitable healthcare delivery and advance our understanding of the genetic basis of human health and disease across diverse populations.

## Materials and methods

### Ethics approval and consents

Approval to conduct the study was obtained from the Institutional Review Board of the Lebanese American University, Beirut, Lebanon (IRB #:LAUMCRH.AM3.2023.R1.1/Feb/2024). All patients or their legally authorized representatives (parental consent) signed an informed consent for participation, sample collection and data publication.

### Studied cohort

Over the last 3 years, 500 patients, seeking genetic diagnosis by ES, were referred to our institution. The studied cohort primarily comprised pediatric patients (representing around 80% of the cohort) seeking genetic diagnosis for various indications including neurodevelopmental disorders, deafness, cardiogenetic syndromes, and family history of inherited conditions). The remaining 20% were young adults seeking genetic counseling prior to marriage.

ES of parents and/or other family members has been performed in a subset of cases when trio or familial analysis was clinically indicated, primarily to aid in variant interpretation and confirm segregation. However, these data were not the focus of this paper and were not included in the summary statistics or secondary findings analysis, which were restricted to probands only.

All those patients, or their legal guardians, wished to receive information on actionable secondary findings, unrelated to the condition for which genetic testing was performed.

### Isolation of genomic DNA, exome sequencing (ES) and variants classifications

Approval to conduct the study was obtained from the Institutional Review Board of the Lebanese American University, Beirut, Lebanon (IRB #:LAUMCRH.AM3.2023.R1.1/Feb/2024). All patients or their legally authorized representatives (parental consent) signed an informed consent for participation and sample collection. DNA was extracted from leucocytes by standard salt-precipitation methods [35].

Exome was captured and enriched using the solution Agilent SureSelect Human All Exon kit version 5.0 and samples were then multiplexed and subjected to sequencing on an Illumina HiSeq 2500 PE100–125. Reads files (FASTQ) were generated from the sequencing platform via the manufacturer’s proprietary software. Reads were aligned to the hg19/b37 reference genome using the Burrows-Wheeler Aligner (BWA) package version 0.7.11 [36]. Variant calling was performed using the Genome Analysis Tool Kit (GATK) version 3.3 [37]. Variants were called using high stringency settings and annotated with VarAFT software 1.61 [38] containing information from dbSNP147 and the Genome Aggregation database (gnomAD, http://gnomad.broadinstitute.org). Only coding and splicing variants were considered. Variant filtering was performed according to the frequency of the variant in gnomAD (less than or equal to 1%; *HFE* was then evaluated independently of the above-mentioned filters, due to the elevated prevalence of certain variants of interest in this gene).

Variants were categorized based on two systems: the ACMG/AMP guidelines and on ClinVar. ACMG is standardized system developed to classify genetic variants based on their clinical significance as outlined by Richards et al. (2015) [6] using a combination of automated tools and manual curation. We initially used InterVar (InterVar-Genetic variants Interpretation by ACMG/AMP 2015 guideline), Franklin (Franklin), and VarSome (VarSome The Human Genomics Community) to obtain preliminary classifications and rule assignments (e.g., PVS1, PS1, PM2, etc.). These automated tools helped identify applicable criteria based on available evidence, including population frequency (e.g., gnomAD), in silico predictions (e.g., SIFT, PolyPhen-2, CADD), and known variant databases (e.g., ClinVar, HGMD). All automated outputs were then manually reviewed and curated by our team of clinical geneticists and molecular biologists. Each criterion was assigned following the strength levels recommended by the ACMG/AMP framework (e.g., very strong, strong, moderate, supporting), and the final classification (pathogenic, likely pathogenic, VUS, etc.) was determined accordingly.

On the other hand, ClinVar is a public database that aggregates interpretations from clinical laboratories and researchers who submit interpretations of genetic variants and the evidence supporting them. In this study, each variant was submitted individually to the ClinVar database, and only the ClinVar-assigned classification was used as well as the associated review status (i.e., the number of stars) to indicate the level of confidence in each classification. Variants that had conflicting interpretations on ClinVar, were considered as variants of “unknown significance”.

## Supporting information

S1 Fig Frequency distribution of depths of coverage (DP) and allele balances (AB). Frequency distribution of DP and AB of all 9632 identified variants with the dashed lines marking the specified thresholds. The plots highlight the expected patterns of DP and AB distributions with no apparent abnormalities. The depth of coverage distribution is shown to have its mode around 40x after which the frequency keeps decreasing. The allele balances of the heterozygous variants vary around a maximum frequency of 0.5, while the balances of the homozygous variants are at the single line at value 1.
(TIF)

S2 Fig Distribution of all ACMG actionable genes and those identified in our cohort across disease categories. The actionable genes identified based on the interpretations of pathogenicity of both ACMG/AMP and ClinVar are displayed for comparison.
(TIF)

S3 Fig Frequencies of mutation types for the identified pathogenic and likely pathogenic variants across phenotypic categories. The findings based on the ACMG/AMP guidelines and ClinVar are separated for comparison.
(TIF)

S1 Table Secondary findings per phenotype category based on the ACMG/AMP guidelines. (XLSX)
