# Supplementary figures and images for: Certain vs. uncertain actionable secondary findings in a cohort of 500 Lebanese participants: What to report to the patient?
Source: PLoS One. 2025 Jul 18;20(7):e0327471. doi: 10.1371/journal.pone.0327471 (PMC12273981; doi:10.1371/journal.pone.0327471)

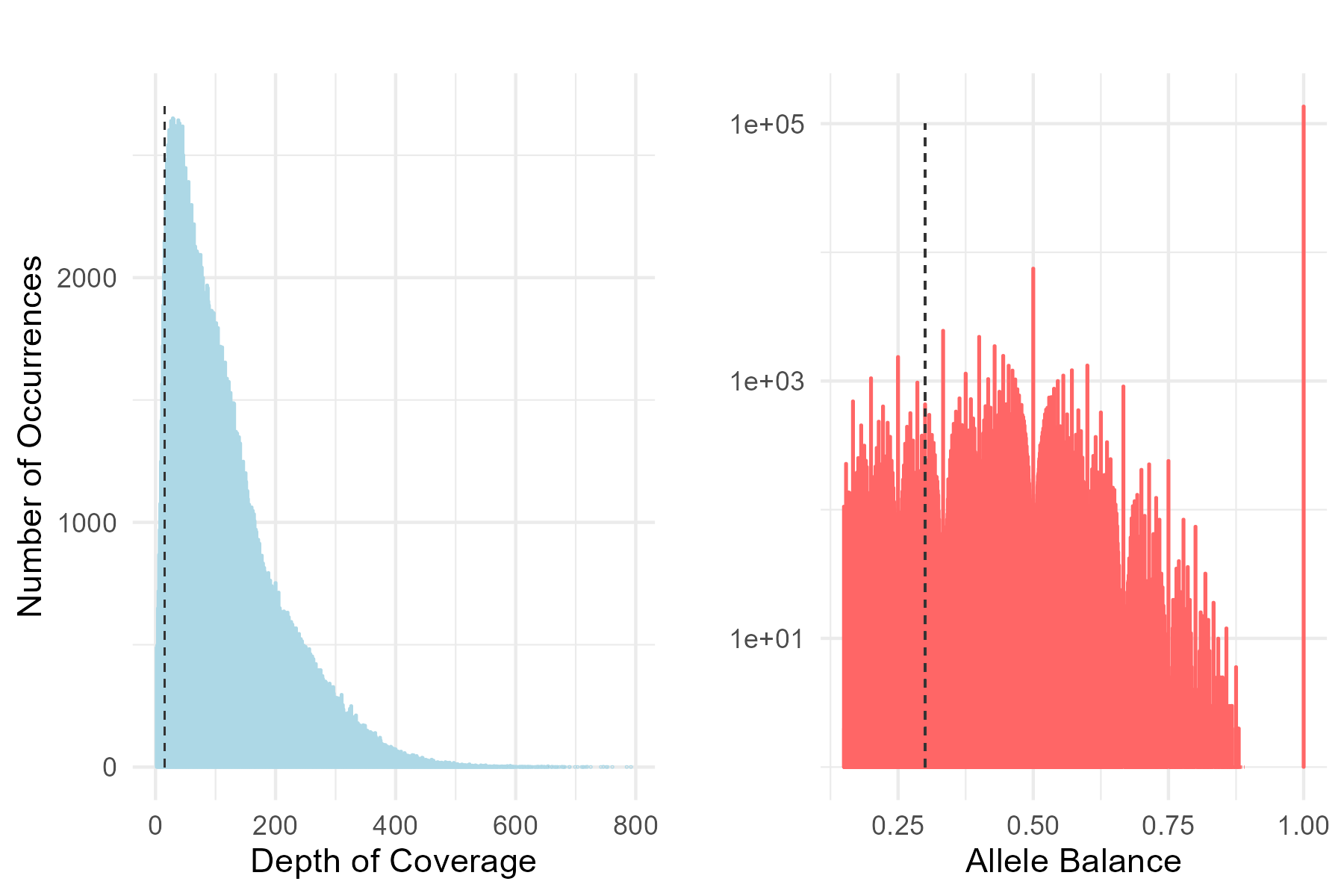

Supplement: S1 Fig — Frequency distribution of DP and AB of all 9632 identified variants with the dashed lines marking the specified thresholds. The plots highlight the expected patterns of DP and AB distributions with no apparent abnormalities. The depth of coverage distribution is shown to have its mode around 40x after which the frequency keeps decreasing. The allele balances of the heterozygous variants vary around a maximum frequency of 0.5, while the balances of the homozygous variants are at the single line at value 1. (TIF) [file pone.0327471.s001.tif]

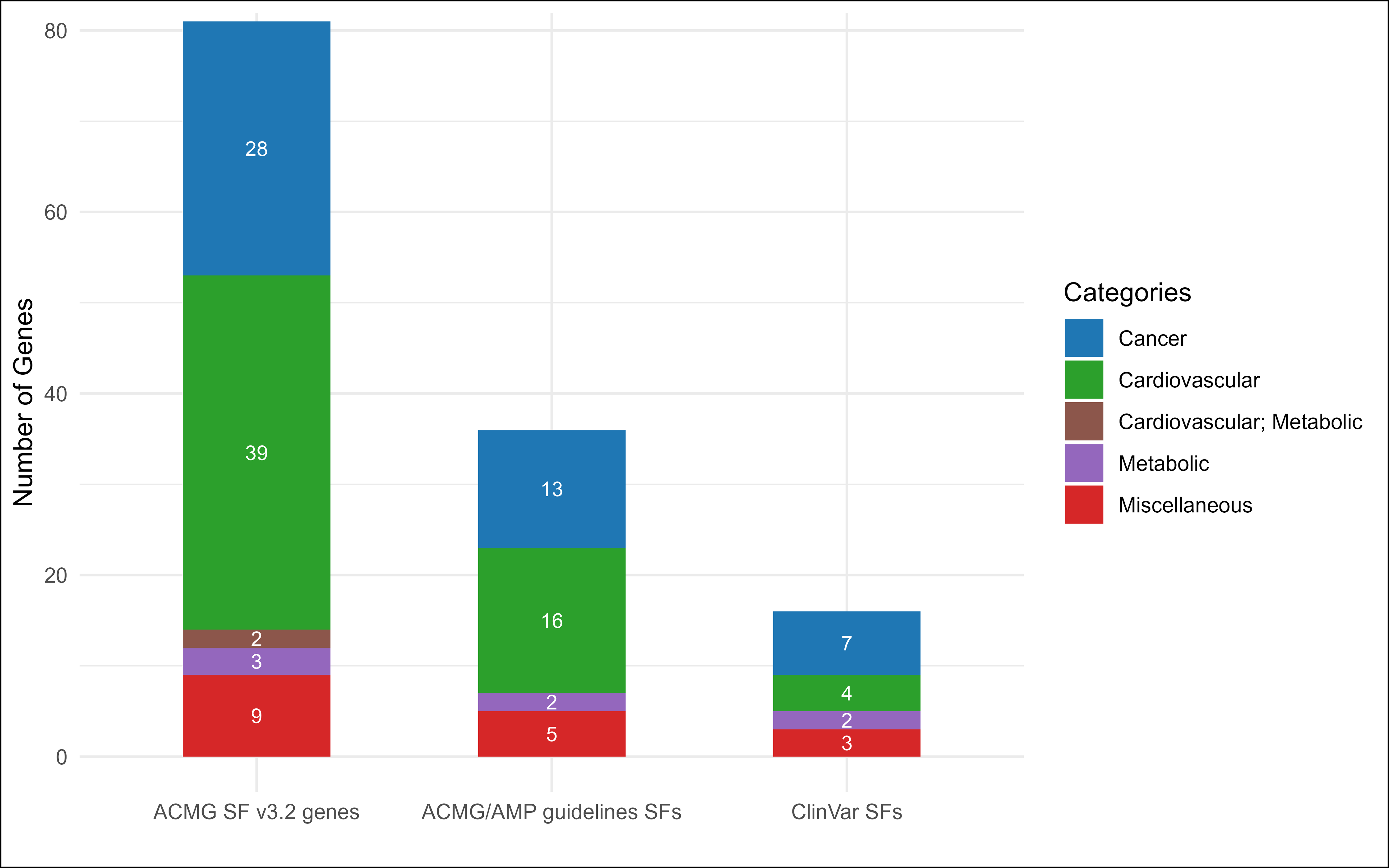

Supplement: S2 Fig — The actionable genes identified based on the interpretations of pathogenicity of both ACMG/AMP and ClinVar are displayed for comparison. (TIF) [file pone.0327471.s002.tif]

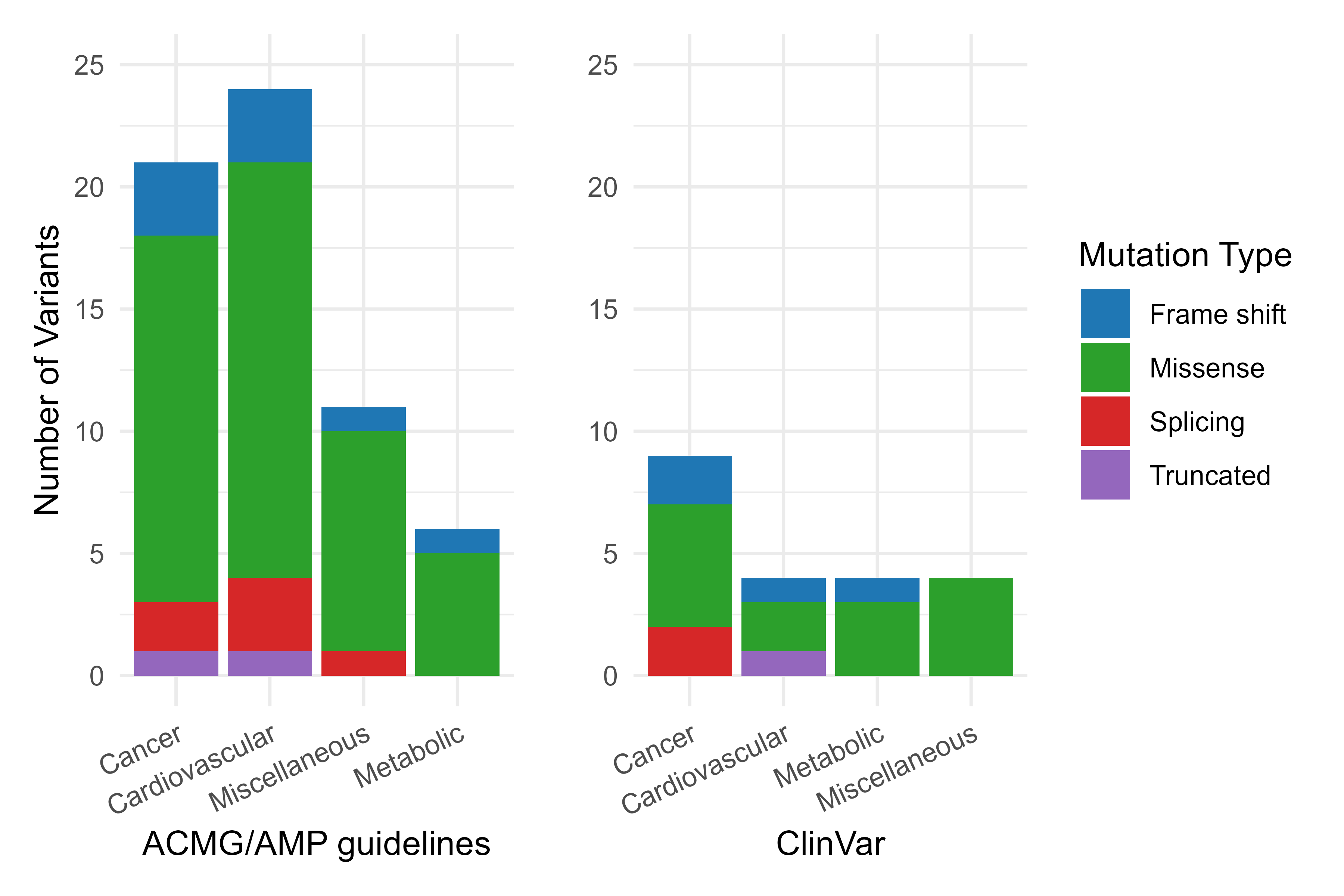

Supplement: S3 Fig — The findings based on the ACMG/AMP guidelines and ClinVar are separated for comparison. (TIF) [file pone.0327471.s003.tif]
